# A Systematic Review of Nutraceuticals from the Perspective of Life-Cycle Assessment

**DOI:** 10.3390/ph18091278

**Published:** 2025-08-27

**Authors:** Ilija Djekic, Nada Smigic, Dubravka Vitali Čepo

**Affiliations:** 1 Faculty of Agriculture, University of Belgrade, Nemanjina 6, 11080 Belgrade, Serbia; nadasmigic@agrif.bg.ac.rs; 2 Faculty of Pharmacy and Biochemistry, University of Zagreb, Ante Kovačića 1, 10000 Zagreb, Croatia

**Keywords:** life-cycle thinking, holistic approach, nutraceuticals, environmental footprints

## Abstract

**Background/Objectives**: Despite its growing application, life-cycle assessment (LCA) in the nutraceutical sector has not been systematically studied, leaving a gap in our understanding of the unique challenges of assessing its environmental footprint. The main objective of this study was to provide an overview of scientific publications related to nutraceuticals from the LCA perspective. **Methods**: This review combined bibliometric analysis, using VOSViewer as an analytic tool, with the search of the Web of Science database, aiming to identify the most relevant papers associated with nutraceuticals and life-cycle assessment. **Results**: The final selection of the most relevant publications was set at 65, analyzing 78 different nutraceuticals. Results reveal that the main sources of raw materials for extraction of nutraceuticals are marine-based, plant-based, and from agri-food waste. Polyphenols were analyzed 34 times and were predominantly sourced from plants, while carotenoids, analyzed 17 times, were mainly linked with marine-based and food waste-derived sources. The main environmental footprints were focused on climate change, covering most of the nutraceuticals analyzed (97.4%), followed by acidification (78.2%) and eutrophication (74.4%). SimaPro was the prevailing software used for 43.6% nutraceuticals, while the prevailing database was Ecoinvent, used in two thirds of the cases (66.7%). ReCiPe, as a life-cycle inventory assessment method, was used for calculating 34.6% of analyzed cases, followed by CML (33.3%). **Conclusions**: This systematic review highlights the main challenge in LCA studies, outlining great variability in study boundaries, functional units, and reported environmental footprints, and making it difficult to compare the environmental impacts of similar nutraceutical groups from a life-cycle perspective. This underscores the urgent need to improve input-data quality and develop standardized methodologies to validate sustainability claims using LCA.

## 1. Introduction

Nutraceutical is a terms that consist of two parts—“nutrition” and “pharmaceutical”—and was introduced by Stephen L. DeFelice in 1989, founder and chairman of the Foundation of Innovation Medicine [1]. Nutraceuticals, derived from natural food sources, offer additional health benefits beyond basic nutrition, including improved physiological functions and protection against chronic diseases [2]. Due to increasing consumer awareness about the relationship between diet and health, the aging population, and the increasing prevalence of lifestyle-related diseases, the nutraceutical market has increased dramatically in recent years. It reached USD 500 billion in 2024, and it is projected to reach USD 878 billion by 2033 [3]. Regional trends show that Asia Pacific leads in market share, while North America remains a hub for innovation and regulatory advancement.

When it comes to purchasing nutraceuticals, consumers rely on various quality cues that share the information about the product to the consumers [4]. Although for some products, such as food, the intrinsic quality characteristics prevail, the extrinsic characteristics that are communicated through labels and advertising (e.g., “immune boosting”, or “eco-friendly packaging”) also play an important role [5]. Therefore, this type of information has a cognitive effect on consumers, influencing how the characteristics are interpreted or understood [6]. This so-called halo effect is defined as a powerful message, raising strong impressions toward a product [7]. In nutraceutical marketing, brands often highlight certain positive features—like being natural or eco-friendly—to create an overall good impression of the product. In general, two main “positive” persuasive messages are associated with two pillars of sustainable diets—“health” and “environment” [8]. “Sustainable diet” is a term developed by the Food and Agriculture Organization, aiming to analyze diets from two main dimensions—their health benefits and environmental impacts associated with the production of food [9]. However, promotion of environmentally friendly production and/or products is sometimes associated with “green washing”, as means of eco-manipulation [10]. This risk is also present in the nutraceutical sector, where environmental or health-related claims are sometimes overstated or unsubstantiated. To combat this issue, it is important to understand what type of environmental information is associated with producers and their products.

The European Union has adopted several legislative measures to strengthen corporate sustainability reporting. Under the Corporate Sustainability Reporting Directive (CSRD), large companies (over 1000 employees) are required to produce detailed sustainability reports, starting from 2024 [11,12]. These reports must comply with the European Sustainability Reporting Standards (ESRS) and include data on climate change, resource use, biodiversity, and social responsibility. In addition, EU regulations also require companies to classify and disclose the sustainability of their economic activities [13,14]. These regulations are important for food and pharmaceutical companies, including the nutraceutical manufacturers, due to their complex supply chains and public health responsibilities. For example, companies are expected to identify and mitigate sustainability risks related to the sourcing of raw materials and production processes [15]. In this context, life-cycle assessment (LCA) emerges as a key tool for evaluating and communicating environmental performance in a standardized and robust manner.

When it comes to products, life-cycle thinking, as a holistic approach, is the basic method that outlines the burdens of products, from raw material extraction to end of life [16]. It is a clearly connected with Sustainable Development Goal #12, “responsible consumption and production”, paving the way to the monitoring of the entire supply chain, including consumers [17]. Life-cycle thinking has started its evolution from Ecolabel Regulation of 1992 [18], bringing us to the latest European Green Deal [19]. LCA is widely used in food and pharmaceutical industries, but its application to produce nutraceuticals remains relatively limited. As production of nutraceuticals relies on resource-intensive production processes, such as extraction, the evaluation of its environmental impacts through life-cycle assessment is very much needed.

Despite the growing interest in sustainable production, to the best of our knowledge, the application of LCA to the nutraceutical sector has not been systematically analyzed. The existing literature addresses food and pharmaceutical products separately, leaving a gap in our understanding of the specific challenges and practices in assessing nutraceuticals.

The aim of this paper is to conduct a review of scientific publications and to examine how nutraceuticals are analyzed from the perspective of life-cycle assessment, focusing on the methods applied, the environmental indicators used, and the consistency of reporting practices. By doing so, this review seeks to clarify how well current research supports the environmental evaluation of nutraceuticals and to highlight where further methodological development or standardization is needed.

## 2. Results

### 2.1. Life-Cycle Assessment (LCA) Background

#### 2.1.1. Fundamentals of LCA

LCA is a concept that evaluates environmental impacts throughout a life cycle of a product, as outlined in ISO 14040 [20]. Despite it being “standardized”, it is a scientific method that analyzes the life-cycle of a selected product within the specified goal of the study, consisting of inventory collecting, calculating, and interpreting environmental impacts holding potential of suggesting improvement measures [21]. Within the defining of the goal of the study, it is important to clarify the scope and boundaries where the boundaries span from “cradle to grave”, with its different varieties, such as “cradle to gate”, covering raw materials acquisition; “gate to gate”, analyzing the production/processing phase; and “gate to grave” investigating the impacts connected with consumers and end-of-life disposal [20]. There are also other suggestions, such as “cradle-to-market” or “cradle-to-use” approaches [21]. Another important issue to be resolved within this stage is the functional unit used for interpretation of results, such as mass, volume, or function of the product. Collecting inventory data may be challenging since data sources vary, raising uncertainties [22]. Data needed for LCA consists of physical inputs (material(s) and energy), products and/or co-products, and all outputs associated with the environment. However, as this stage is considered data-intensive and data-sensitive covering numerous activities, it is of utmost importance to evaluate their influence on overall calculations [23]. The choice of appropriate environmental footprints is the main task in impact assessment [24], where different methodologies deploy them to endpoints such as human health, ecosystems, and resources [25]. A wide range of variables (data sources, methodological choices, system boundaries, etc.) can significantly influence the results of the analysis, leading to considerable variability in data interpretation.

#### 2.1.2. Advanced Life-Cycle Approaches

Acknowledging that sustainability has three main pillars (economy, environment, and society), combined with life-cycle thinking, helped paved the way for developing a Life-Cycle Sustainability Assessment (LCSA) approach, assessing the environmental, social, and economic impacts of a product [26,27]. This assessment is performed using three main tools: LCA, Life-Cycle Costing (LCC), and Social Life-Cycle Assessment (s-LCA) [28]. All three tools follow the same pattern of phases, namely (i) goal and scope definition, (ii) inventory analysis, (iii) impact assessment, and (iv) interpretation, since LCA does not address the economic or social sustainability dimension of a product; however, the life-cycle thinking and approach are applicable for these two dimensions also [20].

LCC converts the production data (i.e., material and energetic flows identified in LCA) into a monetary value, enabling financial cost–benefit analysis of the product [28], while s-LCA is a tool associated with identified stakeholders providing information on the social and socio-economic impacts of a product or organization [29]. The s-LCA is a promising tool that provides valuable information associated with the social dimension of sustainability, but still under development [30]. Although LCC and s-LCA have the same methodological context, they differ in terms of data requirements, collection strategies, limitations and/or exclusions, and impact-assessment indicators [29,31].

### 2.2. Review Process Results

#### 2.2.1. Bibliometric Analysis

**Figure 1:**
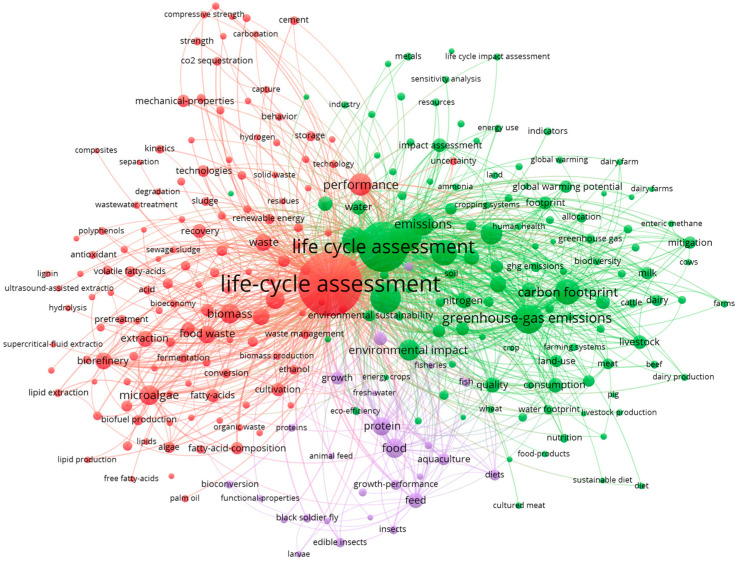
The visual network presentation of the correlation between life-cycle assessment and nutraceuticals.

The results of the bibliometric search are displayed in Figure 1, retrieved from 2979 publications based on the title, abstract, and keywords.

The green “environmental” cluster is mainly associated with LCA and environmental impacts that have been analyzed, such as greenhouse gas emissions, water footprint, energy and resource consumption, or land use. The red “nutraceutical life cycle” cluster is associated with sources of nutraceuticals (such as waste), technologies applied (such as hydrolysis or ultrasound or fermentation), and bioactive components (such as fatty acids, lipids, or polyphenols). The purple “animal-based” cluster is the smallest and is mainly associated with nutraceuticals originating from animal-based sources, such as aquaculture or insects.

This analysis also showed that these papers have been written by 11,816 authors, with 3294 affiliations from 108 countries. The top five countries/regions for the selected period were China, with 386 publications (12.9% of total publications); the USA, with 374 (12.5%); Italy, with 360 (12.1%); Spain, with 249 (8.4%); and Germany, with 232 (7.8%). Out of 2979 publications, 72 were highly cited papers, with one paper reaching over 2000 citations.

#### 2.2.2. Nutraceutical in Focus

**Figure 2:**
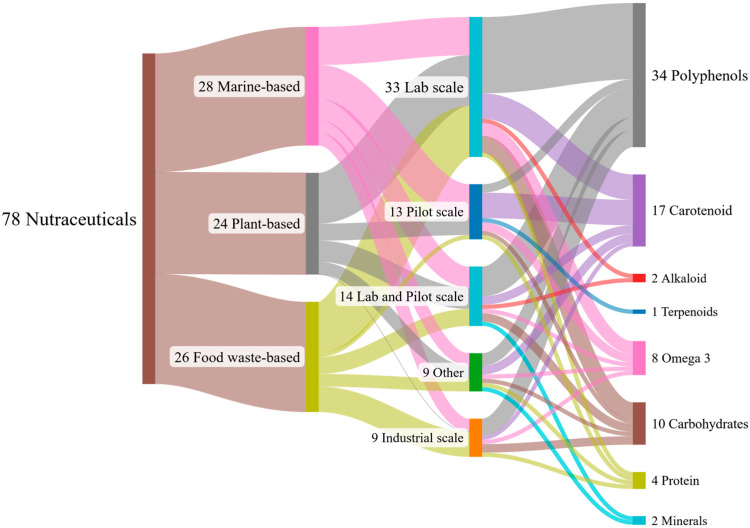
Sankey diagram of 78 nutraceuticals covered in 65 LCA studies.

Table 1 outlines the nutraceuticals deployed, the origin of the raw materials, and the scale of applications and boundaries used in LCA studies. It is supported by Figure 2, depicting the Sankey diagram of the nutraceuticals are the focus of the LCA studies included in this study.

The results show that there were three main sources of raw materials used for nutraceuticals categorized as marine-based, plant-based, and food waste-based. Marine-based sources originate from the sea such as crustacean shells [32], microalgae [33,34,35], or different types of fish [36,37]. When it comes to plants, vegetables [38,39], barks [40,41] and spices [42,43] were prevailing. Finally, the groups of agri-food waste includes the wide variety of sources such as fruit peels [44,45,46] or food processing waste [47,48,49].

Out of 65 reviewed articles, 5 evaluated nutraceuticals belonging to two different groups, and 4 articles assessed nutraceuticals from three groups. This comprehensive evaluation resulted in a total of 78 distinct nutraceuticals included in this diagram. The Sankey diagram (Table 1; Figure 2) shows that polyphenols were mostly derived from plant-based sources and predominantly assessed at the laboratory scale. Carotenoids, particularly astaxanthin and lycopene, were mainly linked with marine-based sources, while functional carbohydrates were derived from food waste.

When it comes to the technologies applied, it is obvious that half of the LCA studies were lab-based (Table 1; Figure 2). A smaller number of studies extended to the pilot scale or combined both laboratory and pilot approaches. Only a limited share of the reviewed studies demonstrated clear industrial applicability. It is of note that our results highlight a significant imbalance in the technology-readiness levels (TRLs), with a drop from lab to industrial applications, particularly for plant-based and food waste-derived compounds. This gap indicates that many environmental impact results are based on conditions that do not represent real production conditions, which can lead to either underestimating or overestimating actual impacts. Without validation at the industrial scale, it is difficult to assess how feasible the process is, or what the real energy use, emissions, and waste generation are in full-scale nutraceutical production. This reveals a gap in upscaling efforts, thus limiting the applicability of environmental findings to real-world nutraceutical production.

In lab-scale studies, extraction was the most used technology, with ethanol as a solvent prevailing [50,51,52], followed by ultrasound-assisted extraction [53,54] or high-voltage electrical discharge. This approach has a scientific explanation, as the justification of the sustainability of non-thermal extraction of bioactive compounds, such as proteins, needs to be narrowed only to the extraction process per se [55]. In parallel, the use of green solvents and promotion of “green” extraction methods can have various benefits, such as higher utilization of the raw material, and lower solvent and energy consumption [56]. However, upscaling the readiness level from lab scale to industrial scale raises sustainability challenges [57], since novel technologies are considered to be “open design challenges”, where the economic and social dimensions need to be considered prior to promoting the technology at an industrial level [58]. It is obvious that the TRL of a technology applied directly influences the understating of its sustainability/environmental impact [59]. Nine TRLs, as defined by the European Commission, have three major milestones—proof of concept, lab-scale application, and full operational/industrial usage [60].

Our analysis of 65 articles shows the range of nutraceuticals included in LCA studies. Among these, polyphenols and carotenoids were the most frequently studied nutraceutical groups, while carbohydrates, omega-3 fatty acids, proteins, minerals, and alkaloids were represented to lesser extents.

The predominance of polyphenols and carotenoids in LCA studies suggests a focus on bioactive compounds derived from agro-industrial by-products and micro-algal biomass (Table 2). These compounds are often present in great amounts in agro-industrial waste, such as fruit and vegetable peels, pomace, leaves, or seeds. Therefore, these compounds are accessible and attractive for valorization using green extraction and circular-economy models. This is in line with sustainability goals, as these studies have a potential to recover high-value antioxidants from waste, and, at the same time, reduce environmental burdens and add economic value to the process. It is of note that polyphenols and carotenoids have a very strong antioxidant capacity [61,62] and therefore may be appropriate for application in the food, cosmetic, and pharmaceutical industries.

Functional carbohydrates and omega-3 fatty acids also received attention due to their recognized health benefits and increasing demand, but their production is often connected with technical and economic challenges [63,64]. It is important to note that numerous studies have focused on the valorization of marine by-products to produce animal/fish feed; however, within the scope of this review, only studies focused on the development of nutraceuticals intended for human consumption were included. This may explain a smaller number of articles covering these compounds. Additionally, dietary fiber is often recovered from food by-products. Despite its common industrial practice, this is considered a lower-value application and may not receive the same research focus in the LCA literature. Additionally, fiber-rich by-products are often used directly in animal feed or compost, which might not require the same level of processing or environmental scrutiny as high-purity extractions of polyphenols or carotenoids.

**Table 2:** Nutraceutical groups, along with sources and extraction methods.

Nutraceutical Group	Sources	Source Type	Extraction Methods	References
Alkaloid	Crambe crambe, Rocoto chili	Marine, plant	Ethanol extraction, encapsulation	[65,66]
Polyphenols	Rosemary, saffron waste, mango waste, citrus waste, onion, wine lees, pomegranate peel, pine needles, moringa, oregano, Ginkgo, tomato leaf, etc.	Plant, waste, marine	Solvent extraction (ethanol, methanol), ultrasound, microwave, Soxhlet, supercritical CO_2_, green solvents, enzymatic, fermentation	[32,40,41,42,43,46,47,48,49,51,54,66,67,68,69,70,71,72,73,74,75,76,77,78,79,80,81,82,83,84,85,86,87]
Carotenoids	*Haematococcus pluvialis*, *Dunaliella salina*, tomato peel, *Phaffia rhodozyma*, algae, carrots	Plant, waste, marine	Ultrasound, solvent-assisted, supercritical CO_2_, bio-based solvents, green extraction	[33,34,35,38,52,53,74,82,88,89,90,91,92,93,94,95]
Functional carbohydrates	Orange peels, grapefruit peels, mango waste, chicory, yacón, chickpea, onion	Waste, plant	Thermosonication, conventional heating, ultrasonication, autoclave, micronization, enzymatic synthesis	[39,44,46,68,71,96,97,98,99]
Protein	Fish skins, Atlantic mackerel, microalgae (*Nannochloropsis*, *Dunaliella*, etc.)	Marine	Enzymatic, NADES, conventional alkali–acid process	[36,37,93,94]
Omega-3 fatty acids	Fish by-products, Phaeodactylum tricornutum, Schizochytrium, microalgae	Marine	Supercritical fluid fractionation, solvent extraction	[65,74,93,94,100,101,102]
Terpenoids	Betulin from birch bark	Plant	Liquid CO_2_ with ethanol	[40]
Minerals and other	Salmon bones, fermentation by-products	Waste, marine	Fermentation, enzymatic hydrolysis	[103,104]

#### 2.2.3. Application of LCA in Nutraceutical Production

**Figure 3:**
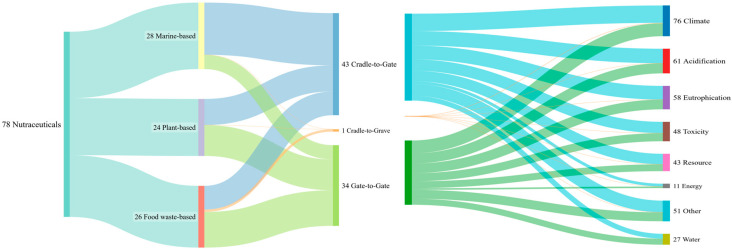
Sankey diagram of LCA boundaries and impacts.

LCA is a concept that evaluates environmental impacts throughout the life cycle of a product. Two main system boundary approaches are commonly used: the cradle-to-gate approach, which includes all steps from raw material extraction to the factory gate; and the gate-to-gate approach, which focuses only on specific stages, such as extraction or formulation. Several LCA software tools are available, including SimaPro, GABI, and openLCA [105,106,107], all of which use databases such as Ecoinvent, ELCD, or GEMIS. Life-cycle impact assessment (LCIA) methods such as ReCiPe, CML, ILCD, and Impact 2002+ are applied to convert inventory data into environmental impact indicators. Figure 3 shows the link between the system boundaries (gate-to-gate and cradle-to-gate approaches) and the environmental impact categories or life-cycle impact assessment (LCIA) methods applied in the reviewed studies. Our systematic review showed that two main approaches were used in LCA studies: the cradle-to-gate and gate-to-gate approaches.

Gate-to-gate studies (associated with 34 nutraceuticals) were mainly conducted at the laboratory scale and focused only on the applied extraction technology by calculating various environmental impacts. In some studies, two or more technologies were analyzed and compared, as in the case of rosemary leaves, where water extraction and particle formation were compared with supercritical fluid extraction and static pressurized hot water extraction [42].

Cradle-to-gate studies (linked with 43 nutraceuticals) were more complex for analysis and are linked with pilot-scale and/or industrial-scale scenarios, as they depend on the raw material source (plant-based, marine-based, and food waste-based) and have different starting points at the “cradle” anchor of the life cycle. They are mainly a combination of in vitro and in silico LCA modelling. In vitro calculations were associated with the extraction process (gate-to-gate approach), while the in silico calculations were used to assess the impacts on the sea, plants, or waste prior to extraction. Finally, only one paper related to one nutraceuticals considered the entire LCA from cradle to grave [104].

Assumptions are often used to simplify the LCA model, resulting in uncertainties that have the potential to significantly affect the results of an analyzed impact [105]. This is one of the main challenges in LCA, as uncertainties are associated with the quality of the data, the uncertainties of the inputs, the variability of data, and the subjective choice of system boundaries [24,108]. Uncertainty analyses are recognized as a promising tool in quantifying different types of uncertainties associated with the LCA model employed, quality of data, depth of inventory analysis, or quality of emission factors [109]. A majority of the studies that were analyzed in this systematic review have not performed uncertainty analyses, which concurs with similar studies [110].

Regarding the software used for calculating LCA, the most frequently used software in reviewed studies was SimaPro (for 43.6% nutraceuticals), followed by GABI (12.8%) and openLCA (11.5%). This is in concurrence with a similar review on LCA of microalgal refinery pointing to these three software [105].

One of the most important parameters is the emission factor, related to a specific inventory; it is directly affected by the databases used. This study reveals several databases used: Ecoinvent, ELCD, LCA Food, IDEMAT, ILCD, and GEMIS. The prevailing database was Ecoinvent, used in two thirds of the cases (66.7%). This is one of the largest and most popular life-cycle inventory databases [111], comprising inputs, outputs, and environmental impacts associated with different processes and activities originating from various industries.

Life-cycle inventory assessment (LCIA) methods used in the analyzed studies were ReCiPe Midpoint/Endpoint, Environmental Footprint, TRACI, IPCC, CML, Impact 2002+, and ILCD. ReCiPe is an LCIA method that calculates emissions and resource extractions and converts them into environmental impacts [60], and it consists of two levels of characterization: midpoint level, with 17 indicators; and endpoint level, with 3 indicators. It was used for calculating 34.6% of analyzed cases. The CML impact assessment method comes from the Netherlands, from the Institute of Environmental Sciences of the University of Leiden [112]. It was used for one third of the cases (33.3%). Environmental Footprint, used in 9.0% cases, is an LCIA method maintained by the European Commission, translating inputs and outputs into environmental-impact categories [113]. ILCD (The International Reference Life Cycle Data) was developed by the European Commission [114] and was used in 5.1% cases. TRACI (Tool for the Reduction and Assessment of Chemical and other environmental Impacts) is an LCIA method developed and maintained in the USA by the Environmental Protection Agency [115]. It was used for calculating 3.8% of analyzed cases. The IMPACT 2002+ LCIA method links different life-cycle inventory results with fourteen midpoint categories to four damage categories [116] and was used in 3.8% of cases. IPCC provides values only for air emissions (and global warming potential) and is developed by the Intergovernmental Panel on Climate Change by the United Nations [113]. The four major LCIA methods in most of the publications are ReCiPe, Impact 2002+, ILCD, and CML [117]; however, not all are updated regularly.

Functional units show the highest diversity in the selected studies, including 1 μg of total intracellular astaxanthin and β-carotene [52,53] associated with the extracted bioactive compound; 1 kg of fish by-product [101] or 2.5 t of processed citrus waste [69] linked with the raw material input; and, finally, one extraction treatment as a functional unit [43,54]. This expresses difficulties in comparing LCA studies with nutraceuticals, as the benchmark differs [24].

Our results indicated that the application of LCA in nutraceutical production is increasing, but studies vary in scope, methodology, and data quality. The major challenges are related to inconsistent system boundaries, diverse functional units, and limited industrial-scale data—factors that complicate comparisons between studies and reduce the reliability of overall sustainability assessments. This also limits the relevance of LCA findings for real-world nutraceutical production, since lab-scale results may not reflect actual emissions, energy use, or feasibility at the industrial scale.

#### 2.2.4. Key Metrics in Nutraceutical Production

The environmental impact indicators extracted from the reviewed studies were categorized into eight main groups, depending on the software used, database applied, and life-cycle impact assessment method applied: (1) climate change; (2) acidification; (3) eutrophication; (4) toxicity; (5) resource use; (6) energy use; (7) water use; and (8) other impacts, such as photochemical smog and ionizing radiation.

The “Climate” group of criteria comprised all global warming potential/carbon footprint values with or without biogenic carbon. In parallel, on some occasions, it included ozone depletion potential/ozone formation. Main indicators are associated with the emission of greenhouse gasses (GHGs) into the air and are expressed as the damage level in kg CO_2_ equivalent (CO_2e_), comprising the weighted impacts of GHGs for the timeframe of 100 years [118]. This environmental impact has been calculated for the majority of the nutraceutical cases (97.4%; Figure 3).

“Acidification” was expressed as terrestrial acidification potential. It calculates the impact of different acidifying substances on the ecosystem, comprising soil, groundwater, and surface water, expressed as kg SO_2_ equivalents [119]. The “Eutrophication” group of criteria had two possible outcomes: either as eutrophication or deployed into freshwater eutrophication, marine eutrophication, and terrestrial eutrophication. It covered impacts associated with the excessive levels of macronutrients in the environment, mainly air, water, and soil, and is typically expressed in kg PO_4_ equivalents [118]. Both acidification and eutrophication were among the majority of environmental impacts (Figure 3). Acidification was covered in 78.2% cases, while eutrophication was covered in 74.4%.

The “Toxicity” group of criteria was the most complex, as it had different toxicity dimensions, such as human toxicity deployed to cancer/non-cancer toxicity, water toxicity (freshwater aquatic ecotoxicity and marine aquatic ecotoxicity), or terrestrial ecotoxicity. It refers to the exposure to and effects of various toxic substances (usually for an infinite time horizon), and was usually expressed as 1,4-dichlorobenzene equivalents (kg 1.4 DB eq) [120]. It was covered in 61.5% of cases.

The “Resource” group of criteria covered the following impacts: fossil resources (fossil resource use and fossil fuel depletion), renewable resource depletion, mineral and metal resource use, land use (something referred to as natural land transformation and urban land occupation), and a generic group named abiotic resource depletion. This clearly depended on the LCIA method used, where different methods focused on different impacts. It was applied in 55.1% cases.

The “Energy” group of criteria was mainly associated with energy consumption and was presented either as cumulative energy demand or primary energy demand, covering 14.1% cases. It analyzed the consumption of different non-renewable and renewable energy sources, usually expressed in MJ [118]. “Water” was mainly associated with water consumption/water use and water footprint, analyzed in 34.6% cases.

“Other” was the final group of criteria that consisted of three types of environmental impacts: photochemical oxidants formation, fine particulate matter, and ionizing radiation mainly originating from ReCiPe and Environmental Footprint LCIA methods.

To understand the existing and future environmental impacts, different modelling scenarios are required, such as upscaling TRL levels [121] with the opportunity to compare it with existing technologies. This is even more pronounced, as “novel” does not necessarily mean environmental friendly, since some LCA scenarios can confirm the opposite [122], such as in a study that confirmed how the supercritical process is more environmentally friendly in relation to energy consumption and GHG emission, but it has a low extraction yield and higher price [88], or when the usage of the ethanol/methane production pathway needed for extraction of astaxanthin had higher environmental impacts, mainly as a result of the consumption of chemicals and energy [89]. This brings us to the fundamentals of LCA and its first step [20]—precise setting of the goal, scope, boundaries, and functional units for upscaling and other LCA scenarios.

### 2.3. Sustainability and Beyond

#### 2.3.1. Green Metrics and Indicators for Sustainability

Recognizing that three sustainability’s pillars—economic, environmental, and social—have driven the creation of advanced life-cycle approaches like LCSA, it is increasingly applied in fields like industrial and environmental sciences, but its use in the fields of pharmaceuticals and food is still limited. This is probably due to the lack of methodological harmonization and comprehensive data availability, which are critical for fully operationalizing LCSA in these complex multidisciplinary fields [123]. These observations are consistent with the findings of this review, revealing that the social sustainability pillar has not been covered at all, while the economic pillar was covered in 18 out of 65 studies (27.7%), mainly as techno-economic studies deployed for cost–benefit and profitability analyses. One study conducted a life-cycle cost approach. These findings highlight an important gap in comprehensive sustainability assessments that should be addressed in future research.

#### 2.3.2. Challenges

This review reveals the need to draft a consensus guideline to validate the claim of nutraceuticals being “green” or “sustainable”, emphasizing the cradle-to-gate boundaries being crucial, as it covers not only the extraction process (gate-to-gate approach) but also spans to the sources of raw materials. In parallel, it would be wise to develop a unique uncertainty analysis method that will enable the benchmarking and comparing of results for the same groups of products [109,110].

This aligns with the need for developing common methodologies when it comes to new, novel, or emerging technologies and approaches, and system transitions [124]. Challenges when performing LCA in the food supply chain are similar in nutraceuticals [24] and comprise the system boundaries of LCA, functional units (volume/mass or extraction runs), quality of data (in vitro or in silico data), and impact assessment (software, database, and LCIA methods are used).

#### 2.3.3. Emerging Opportunities

Despite the abovementioned challenges, several opportunities are emerging that could improve the environmental sustainability of the nutraceutical sector. A key development is industrial adoption of green extraction techniques, such as supercritical CO_2_ extraction, ultrasound-assisted extraction, microwave-assisted extraction, or the use of pulsed electric field [73,78,83]. Energy source and electricity usage remain the critical determinants of overall sustainability. Studies emphasize that the benefits of advanced extraction methods, such as supercritical fluid extraction (SFE), depend heavily on the carbon footprint of electricity and solvent use [41,70]. Moreover, maximizing extraction yield does not always translate to lower environmental burden; simplified processes with minimal chemical inputs can be preferable from a life-cycle perspective [41,88]. Finally, integrating LCA early in process development and utilizing agri-food waste offers further sustainability gains. However, process optimization is still needed, particularly to reduce usage of resources and solvents and to ensure realistic performance at the industrial scale [32,41].

Another important opportunity is the integration of circular-economy principles, particularly through the valorization of by-products and waste streams from food processing [101]. By recovering the valuable nutraceutical compounds from agro-industrial residues, producers can reduce raw material demand and lower environmental burdens, closing the loop on resource use. Renewable energy integration in manufacturing processes presents a significant opportunity to reduce the carbon footprint of nutraceutical production [65].

Finally, striving for standardization of this type of studies and employment of simplified LCA in this field could elevate the sustainability dimension of nutraceuticals, similar to the case of sustainable biofuels [125]. This concurs with some studies emphasizing the benefits of simplification approaches in LCA, providing guidance to researchers and decision-makers [126,127].

These emerging trends highlight a growing alignment of nutraceutical production with sustainable development goals, suggesting that continued innovation in technology and system design will be critical to the development of more sustainable nutraceutical supply chains.

## 3. Materials and Methods

To analyze the application of LCA in nutraceuticals, the authors performed a bibliometric study of publications using VOSViewer (Version 1.6.18) as the analytic tool [128]. The search string included terms related to life-cycle assessment and nutraceuticals. The detailed search string is as follows: ((“life cycle assessment” OR “life-cycle assessment”) AND (“Polyphenol*” OR “Carotenoid*”OR “Fatty acid*” OR “peptide” OR “protein*” OR “prebiotic*” OR “vitamin*” OR “mineral” OR “Antioxidant*”)). This string was applied when using the scientific database Web of Science. As a result, 3150 different publications were selected. To narrow the search, the following predefined inclusion criteria were applied: (i) publications years (2010–2025), (ii) publications in English language, and (iii) only articles and review papers [129]. Finally, 2979 publications were further analyzed. To enable the clustering of the results, the authors applied a cut-off criterion, and, thus, only keywords that were mentioned at least 15 times were considered [130], resulting in a network of nodes and links. The terms mentioned fewer times would have made the map too crowded, while a higher cut-off risk would have caused the exclusion of relevant topics that appear slightly less frequently. Depending on the number of items to be analyzed, the minimum number of occurrences should be at least 5 [128]; however, over 10 enable better visualization [130]. Two rules of the thumb enable interpretation of the figure: (i) “size of the nodes corresponds to the frequency of occurrence”; and (ii) “the width of the links is related to the strength of the connection in-between the terms”.

**Figure 4:**
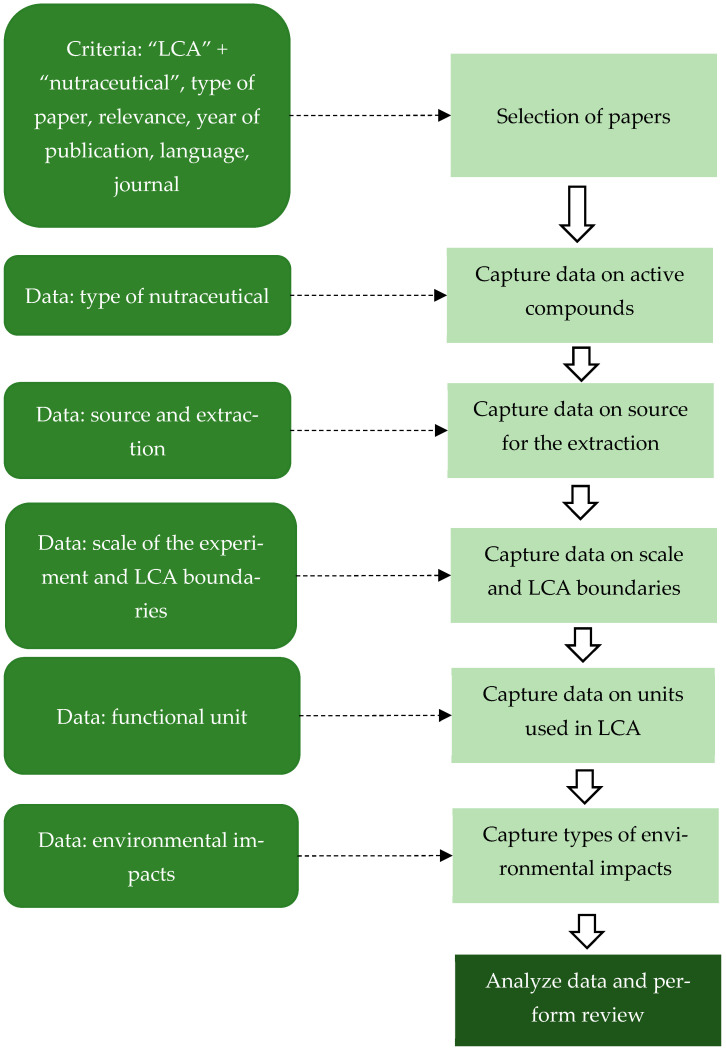
Methodological flow of the review process. Legend: LCA—life-cycle assessment.

To further deploy the search within the Web of Science database, the authors focused on the following two groups of strings: (i) “Polyphenols” or “Flavonoids” or “Phenolic compounds” or “Flavanols” or “catechins” or “epicatechins” or “Flavonols” or “quercetin” or “kaempferol” or “myricetin” or “Anthocyanins” or “Stilbenes” or “resveratrol” or “Tannins” or “Proanthocyanidins” or “Carotenoids” or “Beta-carotene” or “Lycopene” or “Lutein” or “Zeaxanthin” or “Astaxanthin” or “Xanthophylls” or “Retinol equivalents” or “Bioactive peptides” or “Collagen” or “Collagen hydrolysate” or “Lactotripeptides” or “Antihypertensive peptides” or “Casein-derived peptides” or “Whey protein hydrolysates” or “Dietary fiber” or “Prebiotics” or “Inulin” or “Fructooligosaccharides (FOS)” or “Galactooligosaccharides (GOS)” or “Resistant starch” or “Soluble fiber” or “Insoluble fiber” or “Omega-3 fatty acids” or “Eicosapentaenoic acid (EPA)” or “Docosahexaenoic acid (DHA)” or “Alpha-linolenic acid (ALA)” or “Polyunsaturated fatty acids (PUFAs)” or “Fish oil” or “Algal oil” or “Glutathione” or “Coenzyme Q10” or “CoQ10” or “Alpha-lipoic acid” or “Melatonin” or “Nutraceuticals” or “Bioactive compounds” or “Plant-based bioactives” or “Food-derived supplements”, and (ii) “LCA” or “Life-cycle assessment” or “Life cycle assessment” or “Life-cycle analysis” or “life cycle analysis”. This narrowed the search to 367 papers. Exclusion criteria included (i) studies focused on animal feed or veterinary applications, (ii) conference proceedings, and (iii) papers not directly addressing nutraceuticals or life-cycle assessment. The final pool of papers was set at 65, with the following methodological flow of analysis, Figure 4, similar to the work of [131].

## 4. Conclusions

Driven by the growing interest in sustainable production and the necessity to understand potential burdens, the application of LCA in the nutraceutical sector is increasing, enabling a standardized evaluation of environmental performance. In this sector, LCA has predominantly been conducted at the laboratory scale, with fewer studies performed at the pilot scale or combining both approaches. Most available research focuses on the extraction of polyphenols and carotenoids (such as astaxanthin and lutein) from various raw materials, but these studies differ significantly in scope, methodology, and data quality.

Eight main groups of environmental impact indicators (climate change; acidification; eutrophication; toxicity; resource use; energy use; water use; and other impacts, like photochemical smog and ionizing radiation) were identified as the primary focus of the conducted studies. Major challenges that complicate comparisons and reduce the reliability of environmental sustainability assessments include inconsistent system boundaries (cradle-to-gate vs. gate-to-gate approaches). This systematic review underscores the urgent need for consensus guidelines to validate claims of nutraceuticals being “green” or “sustainable”, alongside efforts to improve industrial-scale data quality and develop standardized methodologies.

This analysis identifies three key directions to enhance the sustainability of nutraceutical production: (1) adoption of green extraction techniques; (2) early integration of LCA in process optimization and product eco-design; and (3) incorporation of circular-economy principles—especially through valorization of by-products and waste streams from food processing or integration of renewable energy in manufacturing processes.

This study has four limitations: (i) the systematic review included only manuscripts that were revealed in one scientific database—Web of Science; (ii) potential bias in manuscript selection could lead to a skewed representation of the literature; (iii) risk of being outdated if new research has been published; and (iv) subjective judgments from synthesizing findings outlined in the studies.

## Figures and Tables

**Table 1:** Breakdown of analyzed 78 nutraceuticals in 65 LCA studies.

	Polyphenols	Carotenoids	Functional Carbohydrates	Omega-3 Fatty Acids	Protein	Minerals and Other	Alkaloid	Terpenoids	Total
Total	34 (43.6%)	17 (21.8%)	10 (12.8%)	8 (10.3%)	4 (5.1%)	2 (2.6%)	2 (2.6%)	1 (1.3%)	78 (100%)
Origin of materials used to extract bioactive compounds for nutraceuticals									
Marine-based	3	11	0	8	4	1	1	0	28 (35.9%)
Plant-based	18	2	2	0	0	0	1	1	24 (30.8%)
Food waste-based	13	4	8	0	0	1	0	0	26 (33.3%)
**Scale of applications**									
Laboratory	18	6	4	3	1	0	1	0	33 (42.3%)
Lab and pilot	7	2	2	1	0	1	1	0	14 (17.9%)
Pilot	2	6	1	2	1	0	0	1	13 (16.7%)
Industrial	4	1	2	1	1	0	0	0	9 (11.5%)
Other	3	2	1	1	1	1	0	0	9 (11.5%)
**Boundaries used in LCA studies**									
Cradle to gate	16	11	4	7	3	0	2	0	43 (55.1%)
Gate to gate	18	6	6	1	1	1	0	1	34 (43.6%)
Cradle to grave	0	0	0	0	0	1	0	0	1 (1.3%)

## Data Availability

No new data were created or analyzed in this study.

## Abbreviations

The following abbreviations are used in this manuscript:

LCA	Life-cycle assessment
LCSA	Life-Cycle Sustainability Assessment
LCC	Life-Cycle Costing
s-LCA	Social Life-Cycle Assessment
LCIA	Life-cycle impact assessment
TRL	Technology-readiness level
